# Blood Culture–Negative Infective Endocarditis Diagnosed via 16S rRNA Gene Amplicon Sequencing

**DOI:** 10.1016/j.jaccas.2025.104407

**Published:** 2025-07-30

**Authors:** Masataka Arakawa, Koki Takada, Takahiko Shiba, Takahiko Nagai, Satoru Ogane, Tomoki Shimokawa, Sayaka Katagiri, Takanori Iwata, Ken Kozuma, Akihisa Kataoka

**Affiliations:** a Division of Cardiology, Department of Internal Medicine, Teikyo University School of Medicine, Tokyo, Japan; b Department of Periodontology, Graduate School of Medical and Dental Sciences, Institute of Science Tokyo, Tokyo, Japan; c Department of Plastic, Oral and Maxillofacial Surgery, Teikyo University School of Medicine, Tokyo, Japan; d Department of Cardiovascular Surgery, Teikyo University School of Medicine, Tokyo, Japan; e Department of Oral Biology, Graduate School of Medical and Dental Sciences, Institute of Science Tokyo, Tokyo, Japan; f Division of Oral-Systemic Health, Oral Science Center, Institute of Science Tokyo, Tokyo, Japan

**Keywords:** 16S rRNA gene sequencing, blood culture–negative endocarditis, infective endocarditis, pathogen identification

## Abstract

**Background:**

Infective endocarditis (IE) is typically diagnosed using the 2023 Duke-International Society for Cardiovascular Infectious Diseases criteria, which emphasize positive blood culture results. However, these criteria do not include genome sequencing. The 16S ribosomal ribonucleic acid (rRNA) gene sequencing shows promise for pathogen identification and early detection, particularly in blood culture–negative IE (BCNE).

**Case Summary:**

A 71-year-old man with recurrent fever and dyspnea was diagnosed with IE, causing severe aortic and mitral regurgitation. Although blood culture results were negative, 16S rRNA gene amplicon sequencing identified *Streptococcus* as the causative pathogen. Valve replacement surgery successfully resolved IE and heart failure.

**Discussion:**

Negative culture results were most likely due to prior antibiotic therapy. However, 16S rRNA gene amplicon sequencing revealed a high abundance of *Streptococcus* in the heart, blood, and saliva, demonstrating its diagnostic utility for BCNE.

**Take-Home Message:**

Integrating sequencing technology with bioinformatics advances the diagnosis of IE by enabling early, precise pathogen identification, particularly in BCNE cases.

## Graphical Abstract

**Figure:**
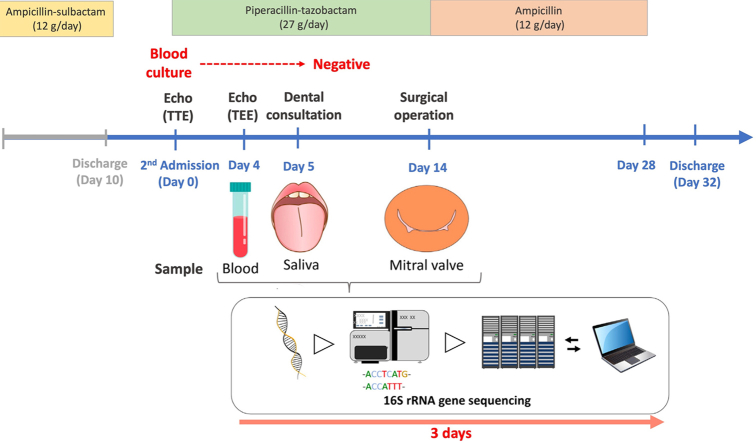

## History of Presentation

**Figure 1:**
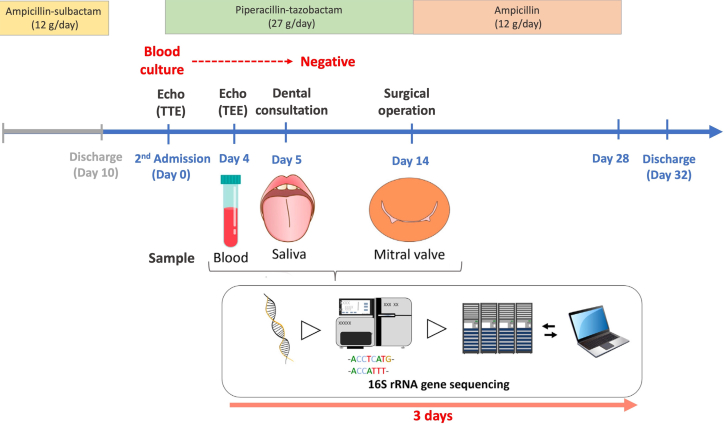
Clinical Course of Investigations and Diagnostics rRNA = ribosomal ribonucleic acid; TEE = transesophageal echocardiogram; TTE = transthoracic echocardiogram.

A 71-year-old Asian man presented with recurrent fever and dyspnea. He was initially diagnosed with pneumonia and acute heart failure, treated with ampicillin-sulbactam (12 g/d), and discharged after 2 weeks of therapy. Twenty days after discharge, the patient was readmitted because of recurrent dyspnea and fever (Figure 1).

**Take-Home Message**

• Sequencing technology combined with bioinformatics may enable the early detection of microorganisms that were previously difficult to identify during the diagnosis of IE, particularly in blood culture–negative IE cases, potentially leading to a re-evaluation of the diagnostic approach using the International Society for Cardiovascular Infectious Diseases IE criteria.

## Past Medical History

The patient had a history of diabetes mellitus and hypertension.

One year prior, the patient underwent tooth extraction and dental caries treatment. At the time of admission, no additional dental treatment was considered necessary. However, details on previous dental procedures were unavailable.

## Differential Diagnosis

Differential diagnoses included recurrent pneumonia, exacerbation of heart failure, infective endocarditis (IE), and other bacterial infections.

## Investigations

**Figure 2:**
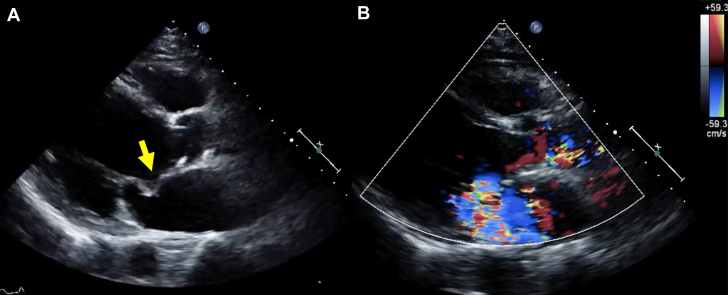
TTE and Color Doppler Image TTE (A) and color Doppler image (B) showing thickening of the anterior mitral leaflet (yellow arrow) and severe mitral regurgitation. TTE = transthoracic echocardiogram.

Hematologic studies on admission showed elevated C-reactive protein levels (12.0 mg/dL) and white blood cell count (18,000/μL), suggestive of bacterial infection. Blood culture results were negative. Physical examination and computed tomography findings provided no evidence suggestive of peripheral embolism. Transthoracic echocardiography revealed severe aortic regurgitation, anterior mitral leaflet thickening, and mitral regurgitation, suggesting IE and acute heart failure (Figure 2, Videos 1 and 2).

**Figure 3:**
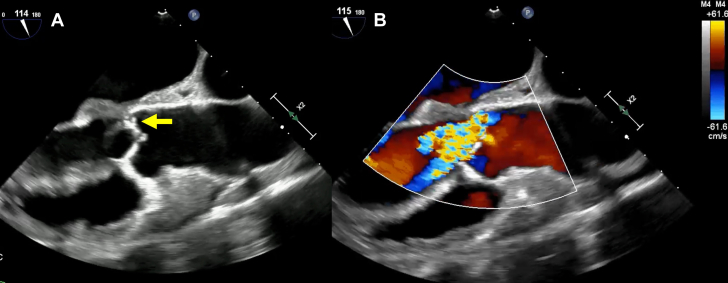
TEE and Color Doppler Imaging of the AV TEE (A) and color Doppler images (B), penetration into the NCC (yellow arrow), and severe aortic regurgitation originating from the NCC and RCC of the AV. AV = aortic valve; NCC = noncoronary cusp; RCC = right coronary cusp; TEE = transesophageal echocardiogram.

**Figure 4:**
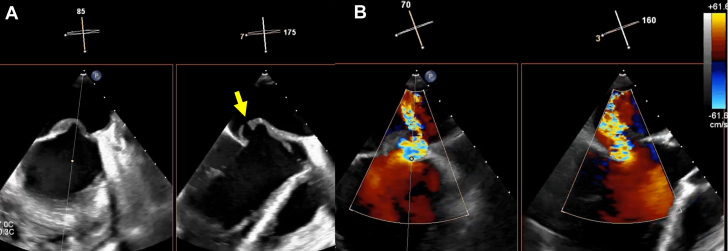
TEE and Color Doppler Imaging of the MV TTE (A) and color Doppler imaging (B) reveal a 3.8 mm perforation and thickening in the A2-A3 segment of the anterior mitral leaflet, accompanied by severe mitral regurgitation originating from the same site. MV = mitral valve; TEE = transesophageal echocardiogram; TTE = transthoracic echocardiogram.

On day 4, transesophageal echocardiography confirmed severe aortic regurgitation originating from the noncoronary cusp and right coronary cusp of the aortic valve (AV) (Figure 3, Videos 3 and 4). A 3.8 mm perforation and thickening were further identified in the A2-A3 segment of the anterior mitral leaflet, accompanied by severe mitral regurgitation originating from the same site (Figure 4, Videos 5 and 6).

In addition, several dental caries were reported. To determine the source of infection, bacterial DNA was extracted from the mitral valve (MV) tissue, blood, and saliva using a DNeasy Blood & Tissue Kit (Qiagen), and the V3 to V4 region of the 16S ribosomal ribonucleic acid (rRNA) gene (341F and 806R) was amplified using Ex Taq HS DNA Polymerase (Takara Bio). The libraries were then sequenced using the MiSeq platform (Illumina) to obtain 2 × 300 bp paired-end reads. The sequence data were uploaded to the DNA Data Bank of Japan under the accession number PRJDB19990. The 16S rRNA gene amplicon sequencing reads were analyzed using QIIME2 (version 2024.2; Gregory Caporaso, Department of Biological Sciences, Northern Arizona University) with Greengenes2 (version 2022.10; Daniel McDonald Department of Pediatrics, University of California San Diego School of Medicine, La Jolla, CA, USA) to investigate the bacterial composition at the genus level.^1^, ^2^, ^3^ Quality filtering and denoising of the sequencing data were performed using the DADA2 pipeline (parameters: p-trunc-len-f: 250 and p-trunc-len-r: 190).

## Management (Medical/Interventions)

**Figure 5:**
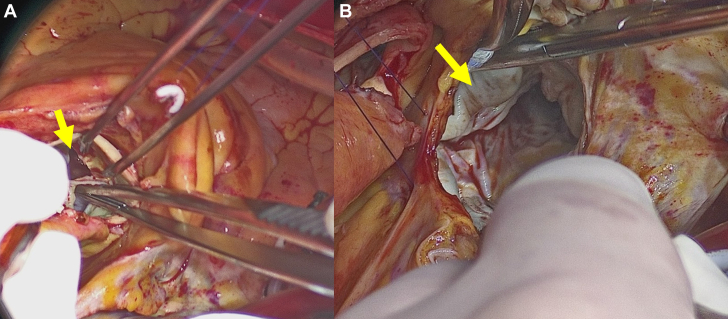
Surgical Findings (A) A perforation was observed in the annulus of the noncoronary cusp of the AV (yellow arrow). (B) The MV shows thickening of the anterior leaflet (yellow arrow), aneurysm formation in the A2 segment, and a partial perforation with suspected vegetation in the anterior commissural area. AV = aortic valve; MV = mitral valve.

**Figure 6:**
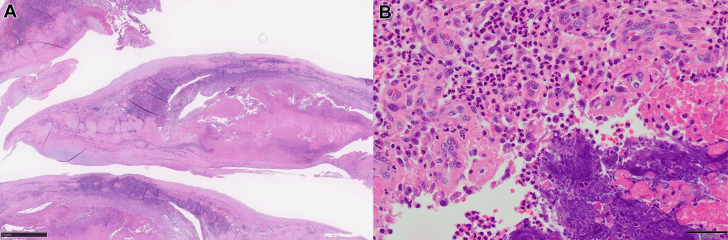
Histologic Images of Mitral Valve (A) Low-power field and (B) high-power field. Histopathologic examination revealed extensive fibrous thickening in the mitral valve accompanied by marked infiltration of inflammatory cells, including neutrophils, within valvular tissue, along with bacterial vegetations. However, no bacteria were cultured from these specimens.

**Figure 7:**
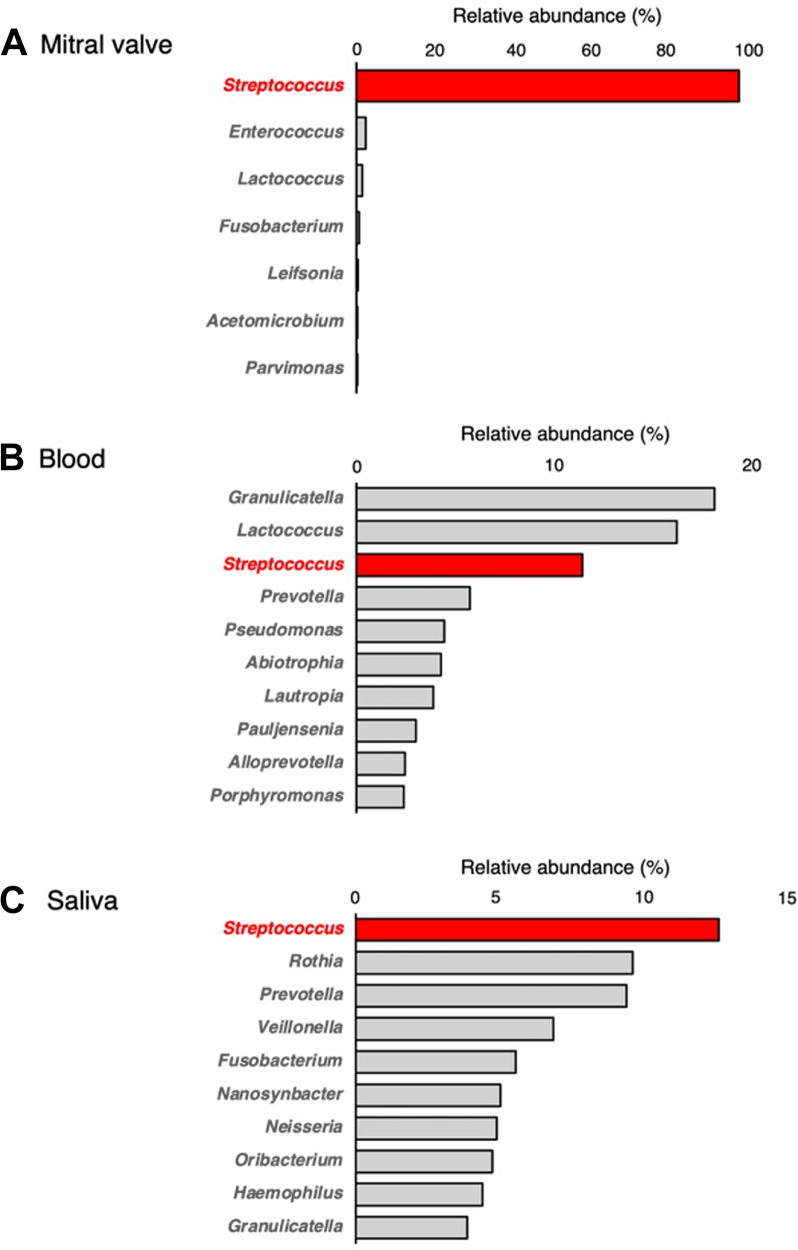
Taxonomic Composition at the Genus Level Taxonomic composition based on relative abundance of sequencing reads at the genus level in (A) mitral valve tissue, (B) blood, and (C) saliva.

On admission, the blood culture results were negative, most likely because of prior antibiotic use. Based on the clinical and echocardiographic findings, empirical treatment for culture-negative IE was initiated with piperacillin-tazobactam (27 g/d). On day 14, quasi-emergency surgery was performed because of progressive heart failure attributed to severe valve destruction. Intraoperative findings revealed a perforation of the annulus of the noncoronary cusp of the AV. MV revealed diffuse thickening of the anterior leaflet, aneurysm formation in the A2 segment, and partial perforation. Vegetation was observed in the anterior commissural area (Figure 5). Both the AV and MV were replaced during surgery. Histopathologic examination revealed extensive fibrous thickening in both AV and MV, accompanied by marked infiltration of inflammatory cells, including neutrophils, within valvular tissue, along with bacterial vegetations. However, no bacteria were cultured from these specimens (Figure 6). Further analysis of the excised MV tissue using 16S rRNA sequencing revealed that *Streptococcus* was the predominant bacterium, accounting for 94.9% of identified genera. *Streptococcus* was also detected in the blood and saliva samples, accounting for 11.1% and 12.6% of the bacterial composition, respectively (Figure 7). Among the top 10 genera detected, *Streptococcus* was the only consistently identified bacterium across all sample types.

Postoperatively, the inflammatory markers improved, and the patient exhibited no symptoms of heart failure, maintaining a stable condition. As part of the postoperative therapy, a 14-day course of ampicillin (12 g/d) was administered. The patient underwent rehabilitation and was discharged on day 32 with no residual symptoms and in good clinical condition.

## Outcome and Follow-Up

Echocardiography at the 6-month follow-up showed no signs of infection recurrence or prosthetic valve complications.

## Discussion

IE is typically diagnosed using the 2023 Duke-International Society for Cardiovascular Infectious Diseases criteria for IE, which include positive blood culture results.^4^ Conventional techniques for blood culture do not always yield an organism; however, a diagnosis of IE can be established if sufficient supporting data are available.^4^ This subset of cases, referred to as blood culture–negative IE (BCNE), poses a significant challenge for clinicians and is associated with increased mortality rates. BCNE can be categorized as bacterial endocarditis with sterilized blood cultures owing to prior antibiotic treatment, endocarditis caused by fastidious microorganisms, or true BCNE caused by intracellular organisms that cannot be cultured using traditional methods. Noninfectious causes such as nonbacterial thrombotic endocarditis should also be considered.^5^ In retrospective studies, the prevalence of BCNE attributed to antibiotic administration before culture varied from 26% to 45%.^6^

In this case, the negative blood culture results were likely influenced by prior antibiotic administration, which occurred 2 weeks before blood samples were collected. In contrast, 16S rRNA gene amplicon sequencing confirmed a high abundance of *Streptococcus* in all tested sites, including the heart, blood, and saliva; notably, this method can also detect DNA from dead bacteria after antibiotic administration. Previous studies have demonstrated that 16S rRNA gene amplicon sequencing accurately identifies bacterial species.^7^

**Figure 8:**
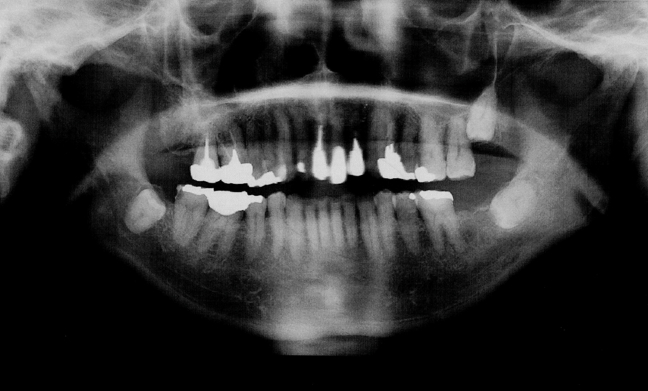
Panoramic x-ray Oral status of teeth with caries-like opacities and dental history of root canal treatment, prosthetic treatment, and tooth extraction.

*Streptococcus* is the first bacterium to colonize the oral cavity after birth and plays a crucial role in regulating the structure and function of the oral microbiota.^8^ Dental procedures, such as tooth extraction and scaling as well as root planning, often involve bleeding, which can allow bacteria to enter the bloodstream and potentially accumulate in the heart valve, leading to IE development.^9^ Approximately 30% of IE cases are estimated to originate in the oral cavity.^9^ Panoramic radiography revealed the oral status with teeth having caries-like opacities and the dental history of root canal treatment, prosthetic treatment, and tooth extraction (Figure 8). These findings suggested that multiple procedures might be implicated in the triggering of infectious endocarditis.

In this study, we used 16S rRNA sequencing, which is primarily recommended for genus-level identification due to its lower accuracy at the species level. Therefore, specific bacterial species and antimicrobial resistance genes were not identified. In addition, the sequencing process took approximately 3 days, with an estimated cost of $100 per sample. Recently, the latest genome sequencing technologies, such as Oxford Nanopore Technologies, have been able to deliver results within 5 to 6 hours of blood collection^10^ at an estimated cost of $50 per sample. These technologies identify pathogens and detect resistance genes, providing accuracy, compared with traditional methods, while significantly reducing the required time.^7^ These advancements are particularly promising for diagnosing BCNE caused by diverse factors, as they enable early detection of pathogens and facilitate the timely selection of effective antimicrobial therapies. This approach could revolutionize the diagnosis and management of IE in the future.

## Conclusions

The 16S rRNA gene amplicon sequencing method combined with conventional blood culture and echocardiographic findings effectively identified pathogens in BCNE cases, even in patients already undergoing antibiotic therapy.

## Funding Support and Author Disclosures

This work was supported by the Japan Society for the Promotion of Science (19K08591 to Dr Kataoka). The authors have reported that they have no relationships relevant to the contents of this paper to disclose.

## Appendix

Video 1 TTE Image Thickening of the anterior mitral leaflet. TTE = transthoracic echocardiogram.

Video 2 Color Doppler Image on TTE Thickening of the anterior mitral leaflet, and severe mitral regurgitation. TTE = transthoracic echocardiogram.

Video 3 The AV on TEE Penetration in the noncoronary cusp on AV. AV = aortic valve; TEE = transesophageal echocardiogram.

Video 4 Color Doppler Image of the AV on TEE Color Doppler image showing severe aortic regurgitation originating from the noncoronary cusp and right coronary cusp of the AV. AV = aortic valve; TEE = transesophageal echocardiogram.

Video 5 The MV on TEE A 3.8 mm perforation and thickening were also identified in the A2-A3 segment of the anterior mitral leaflet accompanied by severe mitral regurgitation originating from the same site. MV = mitral valve; TEE = transesophageal echocardiogram.

Video 6 Color Doppler Image of the MV on TEE Color Doppler image showing severe mitral regurgitation originating from the A2-A3 segment of the anterior mitral leaflet. MV = mitral valve; TEE = transesophageal echocardiogram.
